# Increased burst size in multiply infected cells can alter basic virus dynamics

**DOI:** 10.1186/1745-6150-7-16

**Published:** 2012-05-08

**Authors:** Kara W Cummings, David N Levy, Dominik Wodarz

**Affiliations:** 1 Department of Ecology and Evolutionary Biology, University of California, 321 Steinhaus Hall, 92617, Irvine, CA, USA; 2 Department of Basic Science, New York University College of Dentistry, 921 Schwartz Building, 345 East 24th Street, 10010-9403, New York, NY, USA; 3 Department of Mathematics, University of California, 92697, Irvine, CA, USA

**Keywords:** Multiple infection of cells, Increased burst size, HIV, Mathematical models, Virus dynamics

## Abstract

**Background:**

The dynamics of viral infections have been studied extensively in a variety of settings, both experimentally and with mathematical models. The majority of mathematical models assumes that only one virus can infect a given cell at a time. It is, however, clear that especially in the context of high viral load, cells can become infected with multiple copies of a virus, a process called coinfection. This has been best demonstrated experimentally for human immunodeficiency virus (HIV), although it is thought to be equally relevant for a number of other viral infections. In a previously explored mathematical model, the viral output from an infected cell does not depend on the number of viruses that reside in the cell, i.e. viral replication is limited by cellular rather than viral factors. In this case, basic virus dynamics properties are not altered by coinfection.

**Results:**

Here, we explore the alternative assumption that multiply infected cells are characterized by an increased burst size and find that this can fundamentally alter model predictions. Under this scenario, establishment of infection may not be solely determined by the basic reproductive ratio of the virus, but can depend on the initial virus load. Upon infection, the virus population need not follow straight exponential growth. Instead, the exponential rate of growth can increase over time as virus load becomes larger. Moreover, the model suggests that the ability of anti-viral drugs to suppress the virus population can depend on the virus load upon initiation of therapy. This is because more coinfected cells, which produce more virus, are present at higher virus loads. Hence, the degree of drug resistance is not only determined by the viral genotype, but also by the prevalence of coinfected cells.

**Conclusions:**

Our work shows how an increased burst size in multiply infected cells can alter basic infection dynamics. This forms the basis for future experimental testing of model assumptions and predictions that can distinguish between the different scenarios.

**Reviewers:**

This article was reviewed by RJdeB, RMR and MK.

## Background

The spread of viruses within hosts is characterized by complex interactions among the virus population, its target cells, and immune responses. In addition to experimental work, mathematical models have played an instrumental role in order to elucidate the consequences of these interactions and to understand the dynamics of disease, the development of pathology, as well as the response to treatment [1-3]. While mathematical models of virus dynamics have been applied to several infections, including animal and human viruses, a large amount of work has been performed in the context of human immunodeficiency virus (HIV), where many experimental and clinical data exist that document different aspects of the infection dynamics, allowing parameters to be measured and models to be applied to data sets. Following infection, HIV replicates to high levels during the acute phase, after which the virus typically persists for many years at lower levels during the asymptomatic phase, before finally the immune system collapses and the patient develops AIDS. Crucial kinetic parameters have been measured, including quantification of the turnover rate of infected cells and free viruses [2,4-7], as well as quantification of the basic reproductive ratio of the virus, a measure that determines whether the virus can or cannot establish and maintain an infection in the host [8-10].

Most work on virus/HIV dynamics was performed under the assumption that each cell is infected by a single copy of HIV. It is observed in vitro that upon infection, the virus induces down modulation of its receptor on the surface of the infected cell, rendering the cell resistant to further infection events. It has, however, become clear that multiple copies of HIV can infect the same cell, a process we call coinfection [11-16]. While receptor down-modulation does occur [17,18], it only happens about 24 hours post infection, leaving a time window that allows further copies of HIV to infect the cell [17,19]. Further biological details about the process of coinfection are reviewed in [20]. Coinfection can have important consequences for the evolutionary dynamics of the virus in vivo [16,21-29], for example through recombination between different genotypes that are packaged within the same virus particle. Previous mathematical modeling work has argued that the basic dynamics of HIV infection, including the growth properties in the acute phase of the dynamics, the overall equilibrium properties, and basic drug treatment dynamics, are not altered by the occurrence of coinfection [28,30]. These arguments, however, assumed that an infected cell produces the same amount of virus during its life span independent from the number of viruses that reside in that cell. It can be thought of as virus production that is limited by cellular rather than viral factors [30].

Here, we investigate basic viral dynamics assuming the opposite, i.e. viral factors regulate the extent of virus production such that multiply infected cells produce more virus during their life span than singly infected cells. We find that this can have far reaching consequences for the basic dynamics. Under this assumption, the basic reproductive ratio of the virus alone may no longer be a valid quantity for determining conditions for establishment of infection. Instead, it is found that establishment of infection can also depend on initial virus load, with higher virus loads promoting virus persistence. In addition, it is predicted that the virus population need not follow straightforward exponential growth upon infection. Instead, the exponential rate of virus growth can increase as virus load rises. Finally, whether a virus population responds to treatment or not can now depend on the virus load when treatment is initiated, since the presence of coinfected cells at higher loads leads to overall faster viral replication. The dynamics analyzed by [30] and the ones considered here describe different assumptions about the process of coinfection, which can be addressed by specific experiments. HIV is an obvious system to perform such experiments, although other viruses might be better experimental model systems due to a lesser degree of biological complexity.

## Results and discussion

### A basic model for virus dynamics

This section briefly describes a basic model of virus dynamics in the absence of coinfection. This will provide the basis upon which to build the coinfection models that will be analyzed. The model is based on well-established literature [1,2,31] and includes the following variables: uninfected target cells, *T*, infected target cells, *I*, and free virus, *V*. It is given by the following set of ordinary differential equations that describe the development of these populations over time.

(1) $$\begin{array}{l} \frac{dT}{dt}=\lambda -dT-\frac{\beta TV\left(1+\epsilon \right)}{T+\epsilon }\\ \frac{dI}{dt}=\frac{\beta TV\left(1+\epsilon \right)}{T+\epsilon }-aI\\ \frac{dV}{dt}=kI-uV \end{array}$$

Uninfected cells are produced with a rate λ and die with a rate *d.* Infection by free virus is represented by the term βTV(1+ϵ)/(T+ϵ). The constant *ϵ* appears in the numerator for convenience such that a change in this parameter does not require re-scaling of the parameter *β*. For relatively small values of *ϵ*, this term assumes saturation in the infection term with respect to the number of target cells. For *ϵ*- > 0, the term converges to *βV*. For *ϵ* - > ∞, the infection term converges to *βTV*. Infected cells die with a rate *a* and produce free virus with a rate *k*. Free virus decays with a rate *u*.

The model is characterized by two equilibria. The virus extinction equilibrium is given by the following expressions:

(2) $$T^{\left(0\right)}=\lambda /d;I^{\left(0\right)}=0;V^{\left(0\right)}=0.$$

The virus persistence equilibrium is given by

(3) $$T^{\left(1\right)}=\frac{au\epsilon }{k\beta \left(1+\epsilon \right)-au};I^{\left(1\right)}=\frac{\lambda \beta k\left(1+\epsilon \right)-au\left(d\epsilon +\lambda \right)}{a\left(k\beta \left(1+\epsilon \right)-au\right)};V^{\left(1\right)}=\frac{k\left(\lambda \beta k\left(1+\epsilon \right)-au\left(d\epsilon +\lambda \right)\right)}{au\left(k\beta \left(1+\epsilon \right)-au\right)}\text{.}$$

Whether a successful infection is established or not is determined by the basic reproductive ratio of the virus, *R*_*0*_. It describes the average number of newly infected cells generated by one infected cell during its life span at the beginning of the infection. It is given by $R_{0}=\beta kT^{\left(0\right)}\left(1+\epsilon \right)/\left[au\left(T^{\left(0\right)}+\epsilon \right)\right]$. If *R*_*0*_*>1*, a successful infection is established. If *R*_*0*_ *< 1*, the virus goes extinct. In the following section, we add coinfection to this model.

### Coinfection dynamics without increased viral replication

Basic coinfection dynamics have been described by Dixit and Perelson [30]. By coinfection, we mean multiple infection of cells with different copies of the same virus. Here we adapt model (1) to take a similar form. Instead of a single infected cell population, we now assume the existence of several infected cell sub-populations, i.e. cells infected with i copies of a given virus, *I*_*i*_. The model is given by the following set of ordinary differential equations.

(4) $$\begin{array}{l} \frac{dT}{dt}=\lambda -dT-\frac{\beta TV\left(1+\right)}{T+}\\ \frac{dI_{1}}{dt}=\frac{\beta TV\left(1+\right)}{T+}-aI_{1}-\frac{\beta I_{1}V\left(1+\right)}{I_{1}+}\\ \frac{dI_{i}}{dt}=\frac{\beta I_{i-1}V\left(1+\right)}{I_{i-1}+}-aI_{i}-\frac{\beta I_{i}V\left(1+\right)}{I_{i}+}\\ \frac{dI_{n}}{dt}=\frac{\beta I_{n-1}V\left(1+\right)}{I_{n-1}+}-aI_{n}\\ \frac{dV}{dt}=k\sum_{i=1}^{n}I_{i}-uV \end{array}$$

Cells infected with *i* viruses die with a rate *a*_,_ and infection with an additional virus is represented by the term $\beta I_{i}V\left(1+\epsilon \right)/(I_{i}+\epsilon )$. All Infected cells produce free virus with a rate *k*. This means that infected cells produce the same amount of virus regardless of the number of viruses present in these cells. Hence, virus production is completely determined by cellular resources. Adding more virus genomes to the cell reduces the replicative output of the individual viruses in the cells such that the total amount of virus produced remains the same. Finally, free virus decays with a rate *uV*. Note that the end of the infection cascade, *I*_*n*_, is an artificial feature of the ODE model, and this population should not be concentrated on.

With the exception of some minor differences in some of the terms, this is essentially the model that has been published by Dixit and Perelson [30]. On top of this model, Dixit and Perelson introduced receptor down-modulation, where the permissiveness to further infection declines as a function of time after initial infection. This adds an additional layer of complexity to the mathematical formulation. While this is important for answering specific questions, it does not change the basic dynamics of the system that we are investigating here [32]. In addition, [14] observed no inhibition to virus spread caused by inhibition to reinfection. Hence, for the current purpose we have omitted receptor down-modulation for the sake of model simplicity.

The properties of this model are similar to those of the basic model without coinfection. The reason is the assumption that virus production is regulated completely by cellular factors and that hence, the total amount of virus produced by a cell does not depend on the number of viruses with which the cell is infected. Therefore, the basic replication dynamics are not altered. Again, we observe two outcomes. Virus extinction, given by $T^{\left(0\right)}=\lambda /d;I_{i}{}^{\left(0\right)}=0;V^{\left(0\right)}=0$, and successful establishment of infection, given by the following expressions:

(5) $$T^{*}=\frac{au\epsilon }{k\beta \left(1+\right)-au};\sum_{i=1}^{n}I^{*}=\frac{\lambda \beta k\left(1+\right)-au\left(d+\lambda \right)}{a\left(k\beta \left(1+\right)-au\right)};V^{*}=\frac{k\left(\lambda \beta k\left(1+\right)-au\left(d+\lambda \right)\right)}{au\left(k\beta \left(1+\right)-au\right)}\text{.}$$

The expressions for the individual infected cell subpopulations could not be obtained. However, the sum of the infected subpopulations at equilibrium, as well as the equilibrium levels of uninfected cells and free virus, are the same as in model (1) in the absence of coinfection. Again, the condition for the successful establishment of an infection is provided by the basic reproductive ratio of the virus *R*_*0*_, which is also the same as in the simple model without coinfection.

### Increased viral replication in multiply infected cells

Here we modify the above model to assume that adding more viruses to cells leads to a higher number of viruses produced by an infected cell during its life-span. That is, the burst size of the infected cell increases. We do this by assuming that the rate of new virion production increases as more viruses are added to a cell. All other parameters remain the same. An increased viral output from multiply infected cells can be described as follows: $\frac{dV}{dt}=k\sum_{i=1}^{n}\left(1+\frac{g\left(i-1\right)\left(1+\eta \right)}{i-1+\eta }\right)I_{i}-uV\text{.}$ The parameter *k* describes the basic rate constant for virus production in singly infected cells. Each further virus can potentially add to the rate of virus production in the infected cell. The parameter *g* determines by how much addition of further viruses increases the rate of virus production by the cell. If *g = 1*, an additional virus is expressed independently from the first one. That is, a doubly infected cell produces twice as much virus than a singly infected cell. If *g < 1*, a doubly infected cell produces less than twice as much virus than a singly infected cell. If *g > 1*, a doubly infected cell produces more than twice the amount of virus than a singly infected cell, i.e. there is cooperation between the viruses. As more viruses are added to the cell, however, it would be biologically unrealistic if the rate of virus production steadily increased by the same amount. Hence, a saturation term is included, determined by the parameter *η* (the term *1 + η* in the numerator occurs for the same reason as the term *1 + ϵ* in the infection term described above), The maximum rate of virus production in a multiply infected cell is given by $k\left[1+g\left(1+\eta \right)\right]$.

An increase in the rate of virus production in multiply infected cells could also lead to an increased virus-induced death rate of infected cells. This can be incorporated into the model by expressing the death term as *a*_*i*_. In such a scenario, it would be possible that the burst size of infected cells is not higher in multiply infected cells if the increase in the rate of virus production is offset by an increased death rate of infected cells. Because we are examining the effect of an increased burst size, however, we will assume for simplicity that this comes about through a higher rate of virus production and that the death rate of infected cells will be kept independent of the number of viruses per cell. Note that we also explored alternative expressions to describe this relationship and results did not change on a qualitative level. This, however, will be investigated in a separate paper using more general, axiomatic modeling approaches.

Again, this model is characterized by two equilibria that can be stable. The virus extinction equilibrium, referred to as Equil^(0)^, is given by $T^{\left(0\right)}=\lambda /d;I_{i}{}^{\left(0\right)}=0;V^{\left(0\right)}=0$. The virus persistence equilibrium, referred to as Equil^(^1^)^, is too complicated and cannot be written down. In the following we will examine the condition for successful establishment of infection. Then we will analyze acute infection growth dynamics and equilibrium properties of this model.

In the initial analysis, it will be assumed that the parameter *ϵ* is relatively small, i.e. that the infection term saturates at relatively low numbers of target cells, *T*. This assumption will subsequently be relaxed and discussed.

(a) Conditions for establishment of infection

**Figure 1:**
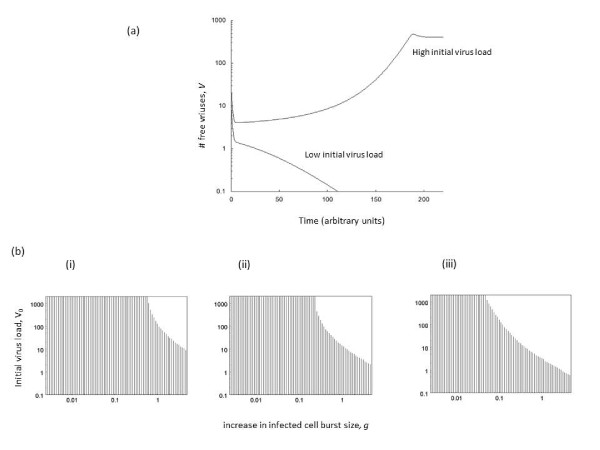
**Dependence of model outcome on the initial virus load.** (a) Two time series that only differ in the initial virus load. In case of the higher initial virus load, the virus population persists. In case of the lower initial virus load, the virus population goes extinct. (b) Graphic showing how the dependence on initial virus load is changed by the degree to which additional resident viruses increase the burst size of infected cells, *g*. In the stripy area, the virus goes extinct. In the white area initial virus load is sufficiently high and a successful infection is established. Panels i-iii show cases with increasing viral infection rates. Parameters were chosen as follows. (a) *λ = 10; d = 0.1; β = 0.02; a = 0.2; k = 4; u = 1; ϵ = 1; g = 0.75; η = 0.5; n = 200. x(0) = λ/d; y*_*i*_*(0) = 0, v(0) = 25 vs 10*. (b) *λ = 10; d = 0.1; a = 0.2; k = 4; u = 1; ϵ = 1; g = 0.75; η = 0.5; n = 200.* (i) *β = 0.015*, (ii) *β = 0.02*, (iii) *β = 0.023.*

In the current model, the basic reproductive ratio of the virus, *R*_*0*_, is not appropriate to determine whether a successful infection is established or not. In fact, *R*_*0*_ is not a meaningful measure in this model. Nevertheless, it is instructive to consider the following related measure: the average number of newly infected cells generated by a singly infected cell during its life span, when placed into a pool of susceptible cells. This corresponds to the basic reproductive ratio of the virus, *R*_*0*_, in a model without increased burst size in multiply infected cells. We call this measure *r*_*0*_. If *r*_*0*_ *> 1*, the infection can be maintained by singly infected cells alone, and an infection is always established in this model. On the other hand, if *r*_*0*_ *< 1*, the singly infected cells alone cannot sustain an infection. However, since viral reproductive output is higher in multiply infected cells, those cell populations can serve as vital sources to establish an infection. Hence, it is crucial that a sufficiently large pool of multiply infected cells is generated at the beginning of the infection. This, in turn depends on the initial virus load. Therefore, if *r*_*0*_ *< 1*, the model can be characterized by bi-stability: both the virus extinction equilibrium, Equil^(0)^, and the virus persistence equilibrium, Equil^(^1^)^, are stable. To which outcome the dynamics converge depends on the initial conditions (Figure 1a). If initial virus load is relatively high, then enough multiply infected cells are generated to establish a persistent infection. On the other hand, if initial virus load is low, then not enough multiply infected cells are generated and the virus population declines to extinction (Figure 1a). Figure 1b shows how the dependence of the outcome on initial virus load depends on the parameter *g*, which determines how fast the rate of virus production goes up with the addition of more viruses to cells. The higher the value of *g*, the lower the initial virus load required to establish an infection. Moreover, the faster the replicative capacity of the virus in singly infected cells (*r*_*0*_), the lower the initial amount of virus required to establish an infection (Figure 1b).

The bistability and the dependence on initial conditions, however, does not always occur if *r*_*0*_ *< 1*. The lower the replicative capacity of the virus in singly infected cells (i.e. *r*_*0*_), the more pronounced the enhanced replication in multiply infected cells has to be for the virus persistence equilibrium, Equil^(^1^)^, to be stable (higher value of *g*). In other words, if the virus replicates slower in singly infected cells, then the addition of another virus needs to increase the overall viral output more for the virus to persist. A detailed stability analysis of Equil^(^1^)^, however, could not be performed because of the complexity of the system. In the context of an increased viral burst size of infected cells, the equilbria could not even be calculated. Analytical work is ongoing in the context of simplified systems which is beyond the scope of the current paper.

So far, no study has investigated whether multiple infection can lead the establishment of infection depending on initial virus load. While multiple infection is best documented with HIV, this issue has not been addressed, although it is documented that successful experimental vaginal infection of monkeys with SIV requires a certain minimum inoculum dose, and that lower dose inoculation can lead to failure to establish the infection, or to transient viremia and subsequent extinction of the infection [33]. Of course, the reasons for this are likely to be multi-factorial, but it is interesting to consider in the context of our model. At the same time, natural HIV infections tends to be initiated with small amounts of virus. Direct cell-to-cell transmission, not included in this model, might be a relevant factor in this respect, or other conditions could enhance the probability of infection (e.g. the presence of wounds or other sexually transmitted diseases). Recent in vitro experiments that study the very early infection dynamics of an adenovirus growing on AD-293 cells are interesting with respect to our findings [34]. This study quantified the extinction probability of the infection depending on how many infected cells are present. As long as the number of infected cells was one or two, a significant extinction probability was observed. If the number of infected cells was three or higher, however, the virus population never went extinct and successful growth was established. This indicates that at this stage, the replication rate of the virus increased sufficiently to prevent extinction. The authors of this study hypothesized that an increase in the number of viruses per cell could lead to faster viral replication which in turn could account for the absence of extinction events. Three infected cells is a rather low number, but this study was conducted using monolayer cultures with agar overlay, which could promote multiple infection of neighboring cells at this stage. These findings could have direct relevance to the dynamics reported here.

(b) Initial viral growth rate during acute infection

**Figure 2:**
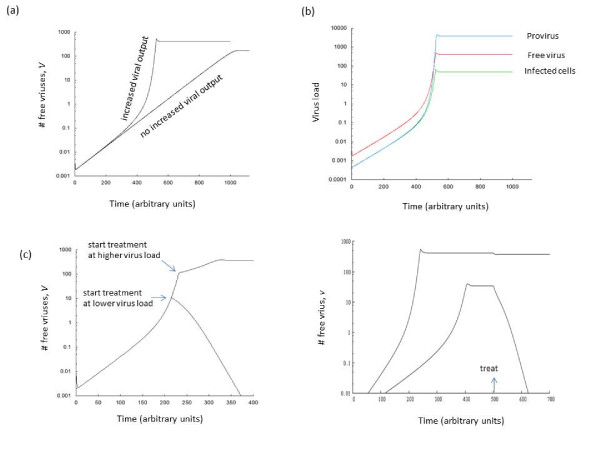
**Effect of increased burst size in coinfected cells on the growth dynamics of the virus population.** (a) Two growth curves are plotted. If no increased burst size is assumed in coinfected cells, then a straight exponential phase of virus growth is observed. In the presence of an increased burst size in coinfected cells, however, the exponential rate of virus growth increases as virus load rises over time. Hence increased viral output in coinfected cells speeds up virus growth significantly. The graph also shows that an increased burst size in coinfected cells leads to a higher equilibrium virus load. (b) This graph shows that for the case of an increased burst size in coinfected cells, different measures of virus load do not necessarily correlate with each other as virus load grows, especially at higher virus loads. This is because the rate of virus production per infected cell increases with higher virus loads. Free virus is given by *v* in the model, the number of infected cells by *ΣI*_*i*_, and provirus is given by the total number of integrated genomes. (c) Increased burst size in coinfected cells and treatment dynamics. Treatment is assumed to reduce the overall viral replication rate to a certain degree. Two curves are shown, where treatment is started at different times. The parameters and the treatment strength are identical for the two curves. If treatment is started earlier when virus load is relatively low, the virus population is suppressed by the drug because the overall rate of viral replication is relatively low. On the other hand, when treatment is started later when virus load is higher, a larger number of coinfected cells with increased viral output leads to faster viral replication kinetics such that the drug is not potent enough anymore to control the virus population. (d) Same principle, but demonstrated in an equilibrium setting. If equilibrium virus load is relatively low, treatment is successful. If equilibrium virus load is higher, treatment can be unsuccessful due to the larger burst size of multiply infected cells. Parameters were chosen as follows. (a,b) *λ = 10; d = 0.1; β = 0.027; a = 0.2; k = 4; u = 1; ϵ = 1; g = 0.75; η = 0.5; n = 200.* (c,d) *λ = 10; d = 0.1; β = 0.03; a = 0.2; k = 4; u = 1; ϵ = 1; g = 0.75; η = 0.5; n = 200. .* During treatment, the viral infection rate was reduced to *β = 0.015.*

Here, we examine the virus growth pattern observed in the model if we start with a relatively low amount of initial virus and let it grow until virus load peaks and converges towards an equilibrium. The growth of the free virus population over time is examined. It is found that the virus population does not grow with a constant exponential rate, as it does in traditional virus dynamics models. Instead, it can be observed that the exponential rate of virus growth increases as virus load grows over time (Figure 2a). The reason is that at low virus loads, most cells are infected with only one virus and the overall rate of virus production in the cells is relatively low. As the virus population grows, an increasing number of cells become infected with more than one virus particle, and this increases the replicative output of the cell. Therefore, the exponential growth rate of the virus population rises. Similarly, starting with a higher virus load results in a faster initial viral growth rate, because more multiply infected cells that can produce a larger amount of virus are generated early on. Note, however, that different measures of virus load (such as free virus, the number of infected cells, and provirus) can show different growth rates as virus growth accelerates at higher loads (Figure 2b).

Data that document the initial growth of the virus have been used previously to estimate basic viral replication parameters, including the basic reproductive ratio of the virus, *R*_*0*_, in the context of SIV and HIV infection [8-10]. The slope of initial virus growth plays an important role in this context, and those data have been fitted with straightforward exponential growth functions. If viral output from infected cells indeed rises in multiply infected cells, then the validity of this approach, and the meaning of the *R*_*0*_, is questionable because the exponential growth rate is not constant over time, but rises. Moreover, these calculations assume that the growth rate of the free virus particles reflects the growth rate of the infected cell populations. If the different measures of virus load show different growth rates, this further complicates the interpretation of the *R*_*0*_ calculations.

It is important to point out, however, that accelerating growth dynamics have not yet been observed in experimental/clinical data from HIV or other infections. Such data typically show straightforward exponential growth. Reasons for this discrepancy need to be investigated. It is possible that growth curves have not been examined in sufficient detail or resolution to see this effect. For example, the acceleration might occur relatively early, before detailed measurements have been taken. Indeed, an accelerating pattern of virus growth has never been specifically looked for in any experimental set-up. Alternatively, it is possible that the rate of virus production does not increase sufficiently in multiply infected cells to observe this effect, or that acceleration only occurs once the number of infected cells has reached very high levels where immune responses induce a slow-down of growth and a decline of the virus population. We note that in our simulations, a pronounced viral peak and subsequent decline in virus load is not observed, mainly because virus-specific immune responses are not included in the model. Hence, immune responses could act in vivo to stop viral growth before the acceleration is observed. Furthermore, the saturation parameter *ϵ* can play a significant role in this respect, which is explored in more detail in Effect of target cell saturation. Finally, other factors might be important which could affect these dynamics. For example, in the context of HIV, recent literature indicates that direct cell-to-cell transmission might play an important role. Previous modeling [32] suggests that under cell-to-cell transmission and in the context of spatially restricted virus spread, multiple infection can significantly drive the dynamics even at the lowest virus loads, which could influence the results. This is under investigation in a separate study. In addition to HIV, these dynamics could be relevant to other viruses in which multiple infection occurs, such as the adenovirus system described above [34].

(c) Drug treatment

In traditional virus dynamics models, the basic reproductive ratio of the virus, *R*_*0*_, is thought to be an important measure for quantifying by how much anti-viral treatment has to reduce the rate of viral replication in order to achieve successful virus control. If drug therapy is strong enough to push *R*_*0*_ below unity, then treatment is predicted to be successful, independent from the virus load at the start of treatment. The current scenario paints a more complicated picture. The strength of the drug required to lead to a sustained decline in the virus population depends on the virus load at the start of therapy. The higher the virus load, the larger the number of multiply infected cells, and the higher the overall replication rate of the virus in the infected cell population. Thus, while a drug can lead to sustained suppression of the virus when treatment is initiated at relatively low virus loads, it might fail when treatment is initiated at a higher virus load (Figure 2c, d). This also has implications for the definition of drug resistance. While drug resistance can be defined in a variety of ways, let us define it by the ability of the drug to drive the virus extinct in the model (note that virus extinction in the model corresponds to sustained drug-mediated control in reality because the model lacks complicating factors such as latent reservoirs that prevent virus eradication). The virus can be called sensitive if the drug can drive the virus extinct in the model. Otherwise, the virus is called resistant to the drug. In this case, the same virus can be resistant to the drug when present at relatively high numbers, while it can be sensitive to the drug at relatively low abundances (Figure 2c,d). Thus, drug resistance can be a function of the presence of coinfected cells. This has also been suggested by [35]. This study, however, argued that this is simply due to the increased number of infection events that can occur when multiple viruses enter a cell through the virological synapse [36]. In our model, the decreased drug sensitivity of the virus population in the presence of coinfected cells is caused by the higher burst size in such cells.

(d) Equilibrium properties

**Figure 3:**
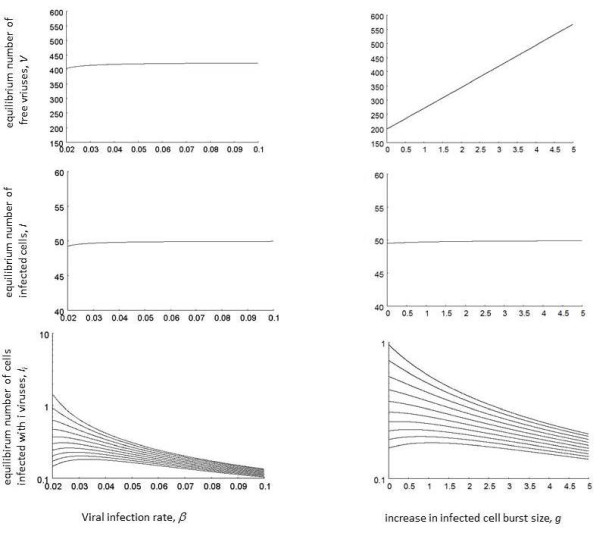
**Effect of viral replication parameters on equilibrium virus load in the model with increased burst size in coinfected cells, expressed as different measures: free virus, the number of infected cells, and different subpopulation of infected cells.** We plotted cells infected with 1, 10, 20, 30 40, 50 60, 70, 80, 90 viral copies, going from the top curve to the bottom curve. While the average number of viruses per cell has been reported to be around 3–4 in the context of HIV replication in tissues, cells infected with a significantly higher copy number have been reported to exist in HIV. See text for details. Base parameters were chosen as follows. *λ = 10; d = 0.1; β = 0.05; a = 0.2; k = 4; u = 1; ϵ = 1; g = 0.75; η = 0.5; n = 200.*

Many of the equilibrium properties of this system are consistent with traditional virus dynamics models that do not take into account coinfection or an increased burst size of multiply infected cells [1]. However, we briefly discuss the equilibrium properties of the individual infected cell subpopulations *I*_*i*_ as a function of the viral spread rate. While the viral spread rate is influenced by several parameters, we focus on the rate of virus infection, *β*, and the degree to which the viral replication rate is increased in multiply infected cells, *g* (Figure 3). The number of cells infected with one virus, *I*_*1*_, declines as the rate of viral spread is increased. The reason is that with a higher rate of virus spread, more cells become infected with multiple viruses. Cell populations that contain multiple viruses can first show an increase for higher viral spread rates and then a decrease. They first increase because more infection generates a higher number of these cells. They subsequently decrease because even more infection events means that the cells become infected with an even higher number of viruses. 

### Effect of target cell saturation

We employed well-established models [1,2] to study the effect of increased viral replication in multiply infected cells on coinfection dynamics. One of the model parameters, however, remains more uncertain than the rest. The rate of virus infection is a saturating function of the number of target cells, and the number of cells at which this saturation occurs is determined by the parameter *ϵ*. So far, we assumed that *ϵ* is relatively small. If the value of *ϵ* is large, saturation practically does not occur and the model properties approach those of simpler models with unsaturated infection terms. The larger the value of *ϵ*, the more the amount of virus production needs to be increased in coinfected cells in order to observe the altered dynamics described here. For large values of *ϵ*, the fraction of coinfected cells becomes vanishingly low during the initial growth phase of the virus. Hence, the dynamics are essentially only driven by singly infected cells. The reason is as follows. At the initial stages of the infection, the number of uninfected cells is significantly larger than the number of singly infected cells. Hence, the number of singly infected cells generated is significantly larger than the number of singly infected cells that are lost to give rise to doubly infected cells. Thus, doubly, and multiply infected cells, make up a relatively small fraction of the total infected cell population. With lower values of *ϵ*, when saturation of the infection term occurs at lower target cell numbers, the difference in the generation and loss of infected cells *I*_*i*_ becomes less, leading to a higher fraction multiply infected cells that can contribute more to the dynamics. In experiments with HIV where the dynamics of coinfection was studied using reporter viruses with different colors [14], it was observed that cells infected with viruses of two different colors can show a prevalence of >40% during virus growth, which is an underestimation of the true fraction of coinfected cells and which is difficult to account for with a large value of *ϵ*. This supports the notion that at least in vitro, low values of *ϵ* could be more realistic. No data are available that quantify the percentage of multiply infected cells during initial virus growth in vivo, and this likely depends on the location of viral replication. In chronic infection, on average 3–4 HIV copies are found per cell in the lymphoid tissue [13], while most infected cells in the blood contain a single copy of the virus [37]. Specific experiments should be designed to estimate the parameter *ϵ* by fitting mathematical models to basic virus growth curves.

## Conclusion

In this paper, we have made the assumption that the burst size of infected cells (amount of virus produced by a cell during its lifespan) can increase with the number of resident viruses. According to our model, this assumption can significantly change the basic properties of virus dynamics, especially if the infection term is a saturating function of the number of target cells. The condition for successful establishment of infection is not determined solely by the basic reproductive ratio of the virus anymore. In fact, the basic reproductive ratio of the virus becomes difficult to interpret, since the different infected cell sub-populations are characterized by different replication rates. This adds to previous theoretical work that have found caveats with regards to the concept of the basic reproductive ratio of the virus, e.g. [38,39]. We found that even if replication in singly infected cells is too slow to initiate sustained virus growth, a successful infection can still be established if the initial inoculum dose is sufficiently high, leading to the immediate generation of coinfected cells that are characterized by a higher burst size. The model further suggests that acute viral growth does not follow straightforward exponential growth, but that the exponential rate of virus growth accelerates as virus load rises. Similarly, virus growth is faster for higher inoculum doses. Finally, the model suggests that the same virus type can be resistant to drugs when present at high loads, while it can be responsive to drugs at lower virus loads. Higher virus load means more coinfected cells, leading to an overall faster rate of viral replication. Hence, the degree of drug resistance depends on the presence of coinfected cells in this model.

An important experimental step will be to quantify the burst size of infected cells depending on the number of resident viruses in specific systems. If the burst size of infected cells does increase if more viruses are present in the cell, this should be investigated in more depth. We assumed that the burst size approaches an asymptote as the number of viruses in the cell increases and the ability of the cell to generate new virions saturates, which is a reasonable assumption. However, the general results derived here are unlikely to depend on this detail, as long as adding viruses to a cell can lead to an increased viral output during the life-span of the cell. A possible scenario that has not been explored in our models is the case where the number of viruses produced by a cell during its lifespan first increases as more viruses are added to the cell, but then decreases again when the number of viruses per cell rises further. It is feasible that the presence of too many resident viruses disproportionately increases the toxicity for the cell, which could outweigh any increased rate of virus production, thus limiting the overall burst size of the cell. However, this is unlikely to have a significant effect on the dynamics because cells containing more viruses would have to reach a sizable proportion of the total infected cell pool. If coinfected cells drive the dynamics, the contributions most likely come from doubly infected cells as these are the most abundant population of coinfected cells. However, the occurrence of direct cell-to-cell transmission in the context of HIV, which can involve the transfer of a large number of viruses per cell [36], has to be kept in mind in this context.

It is so far not possible to reject or confirm the validity of the dynamics described here in specific experimental systems. With regards to HIV infection, previous modeling approaches that do not take account of coinfection have been used describe HIV dynamics and were able to fit data well, and it can thus be argued that a burst size that is independent from the multiplicity of infection is the correct assumption. In addition, as mentioned above, specific predictions such as accelerated virus growth have not been observed in data so far. While reasons for this have to be investigated (see Increased viral replication in multiply infected cells), it is not possible to conclude that the dynamics described here do not occur, since this has never been specifically examined. Thus, new experiments have to be performed that directly test the assumptions and predictions of the model. To test model predictions, in vitro experiments could be used to infect a culture of target cells with a range of different initial HIV doses, and to examine whether higher initial doses lead to a sufficient number of coinfected cells and a consequent faster initial virus growth. Similar experiments can be performed in other virus-cell systems where multiple infection is thought to play an important role, such as in adenoviruses.

In summary, we have explored the opposite assumption about coinfection compared to previous modeling approaches, i.e. that the burst size increases in multiply infected cells rather than being independent of the number of viruses with which a cell is infected. This assumption gives rise to significantly altered virus dynamics that we have described here. The first step will be to test this assumption in specific experimental systems. If it is indeed confirmed that multiply infected cells are characterized by an increased burst size, the model predictions derived here need to be tested. If specific predictions are not confirmed experimentally, other factors might be influencing the dynamics that have not been taken into account in our basic model. In this case, the model and potential modifications can be valuable to identify those factors and thus to improve our understanding of the infection dynamics. Among the experimental systems, HIV has the advantage that multiple infection has been clearly demonstrated to occur and that it has been investigated in more detail than in other viruses. The disadvantage is the complexity of this infection, which leaves uncertainties regarding the exact assumptions that should enter the model. Therefore, it would be insightful to use simpler experimental systems where multiple infection is thought to occur in order to explore the model predictions presented here. Comparing the coinfection dynamics across different virus-cell systems might further shed light onto the validity of the model predictions described here and the circumstances under which they do or do not hold.

## Methods

The work is based on systems of ordinary differential equations that describe the average time evolution of cells and viruses. The equations are specified in the appropriate places in the main text. They were analyzed by a combination of analytical and numerical techniques. The analysis of the multiple infection model assuming an increased burst size of coinfected cells was studied mostly with numerical simulations due to the complexity of this model. It was not possible to specify the equilibrium expressions for this model, and standard stability analysis of the virus persistence equilibrium could not be achieved. In a separate study, we are investigating simplified versions of this model in order to gain more analytical insights.

## Reviewers’ comments

### Reviewer 1- Prof. Rob J de Boer

“The authors study the effects of coinfection of single cells with several copies of the same virus, considering scenarios where the burst size increases with the number of virus particles in the cell. Coinfection has been studied before, but this scenario with an increased burst size is new, interesting, and potentially true. The paper is well written. One the one hand one could argue that allowing for a positive feedback in the burst size leads to a faster than exponential growth is an expected result. On the other hand, I was not certain of some of the results, suggesting that it would indeed be worthwhile to study a model like this. One general question I asked myself is the case where having n viruses in one cell would just increase the burst size n-fold. My intuition tells me that one should then have no positive feedback, i.e., just exponential growth, because in such a system there would just be less target cell limitation as infected cells remain to be equally functional target cells. Given the rather complicated saturation functions in the model, which could even be wrong (see below), I find this simple case hard to find back in the results. I would recommend to help the reader to develop an intuition for the model by explicitly discussing this case.”

**Author response:** At the beginning of virus growth, when virus load is relatively low, the number of multiply infected cells is negligible compared to the number of singly infected cells. Mostly, singly infected cells are generated. Hence the growth rate of the virus population is determined by the rate of virus production in singly infected cells. When virus load becomes higher, multiply infected cells make up a higher fraction of the infected cell pool and their contribution will become visible. Because the rate of virus production in the multiply infected cells is higher than in singly infected cells, you will see the acceleration. This acceleration will be more pronounced in the quoted simple case (just increasing the burst size n-fold) compared to a lesser increase in the burst size, because the rate of viral replication in multiply infected cells is faster. We have tried to express this more clearly in the text. We have also added text discussion the conditions that promote acceleration in growth.

“In several places I find that the equations do not match with what is written in the text. This markedly complicated the reading and prevented me from fully understanding the paper.”

**Author Reply:** we have corrected mistakes that were present in the original version. Thank you for pointing them out! There was also a problem with PDF conversion that messed up some equations, although we are not sure whether this contributed to the problem or not.

Specific comments:

“On page 4 you write "On the other hand, previous mathematical models", but where is the "First hand"?”

**Author reply:** corrected.

“On page 5 you write "can be addressed by specific experiments". Please elaborate: which experiments are you proposing?”

**Author reply:** we have now suggested experiments in the conclusion section.

*“In Eq1 the saturation term is*$\beta T\;v\left(1+\varepsilon \right)/\left(T+\varepsilon \right)$*. In the text below the Eq you write that infection occurs at a rate*$\beta T\;v/\left(T+\varepsilon \right)$*. The latter I understand. The former would allow for infection in the absence of virus.”*

**Author response:** First, the typo in the text has been corrected. It now reads $\beta T\;v\left(1+\varepsilon \right)/\left(T+\varepsilon \right)$. Regarding the nature of this infection term, please note that the factor (1+ε) in the numerator is nothing but a constant, which does not change properties of the model. A simple rescaling of $\beta ->\beta ’=\beta \left(1+\varepsilon \right)$ brings the term to its more familiar form, $\beta ’T\;v/\left(T+\varepsilon \right)$. The factor (1+ε) was added for convenience , such that the term has the following intuitive limiting cases: (i) when ε -> 0, we have βTv/T and (ii) when ε -> ∞, we have βT v (no saturation). Without (1+ε) in the numerator the latter limit would be zero and require a rescaling of β. Note, however, that the presence of (1+ε) in the numerator does not change biology: when v=0, the infection term is zero.

“A minor point: you write that cells die at a rate dT and aI, but each cell dies at rate d or a. Finally, why use a for a death rate, and why two variables in capitals and one in lower case?”

**Author response:** regarding first point, it was corrected. We used “a” for the infected cell death rate to differentiate it from the uninfected cell death rate, “d”. We have capitalized “V” throughout the paper for consistency.

“Eq2: please explain that the model is about identical viruses.”

**Author response:** done.

“On the top of page 6 you write that infected cells are infected at a rate βIiv, but in Eq2 infection of Ii cells happens with the strange saturation term.”

**Author response:** corrected.

“On page 7 you write that infected cells die at a rate aiIi whereas in Eq2 this is aIi.”

**Author response:** corrected.

*“On page 8 you introduce an even more complicated saturation term, which could be suffering from the same problem with a (1 + η) in the numerator. Now it is more difficult to see whether this is a bug or a feature (I vote for a typo). Why not use a conventional Hill function, and write something like*$k\sum_{i}Ii\left(1+g\frac{i-1}{i-1+\eta }\right)$*for the production term? This would have a maximum production of k(1 + g) and be half maximal increases*$k\left(1+g/2\right)$*when i-1 = η.”*

**Author response:** Please note that the Hill function mentioned by the reviewer differs from our term by a constant factor (1+η). Therefore, there is no difference between our function and the one suggested. Please see our response about factor (1+ε), which explains the reasons behind using this constant.

“Page 9: Whether or not an infection gets established in the first phase should hardly depend on these effects because an infection is expected to start with a limited number of virus particles and probably hardly any coinfection. Shouldn'tcoinfection not be something happening at higher virus loads?”

**Author response:** The applicability of the initial condition dependence is now discussed. First, as mentioned in the text, it only occurs if the basic reproductive ratio of singly infected cells is less than one. If the replication rate is faster such that the infection could be established based on the kinetics in singly infected cells, establishment of infection is independent of initial conditions. Having said that, however, we think that the initial condition dependence is still an intriguing result that could have relevance to specific infections. HIV is discussed a lot in the paper. While there is no direct data that examines possible dependencies on initial conditions, it could be hypothesized that this contributes to the relative inefficiency of transmission of the virus to a new host. In order to ensure infection of monkeys with SIV in laboratory settings, a certain threshold infection dose has to be applied. Of course this does not show that the dynamics described here are at work, but it does point to a certain dependence on initial conditions. In addition, we recently examined very early events of viral spread using adenovirus infection of 293 cells in vitro (Hofacre et al. 2012, Virology 423, 89-96). The interesting finding was made that there is a high chance of virus extinction while only one or two infected cells were present, but that no extinction occurs once three infected cells exist. It appears that once thee infected cells exist, the MOI is sufficiently large such that multiple infection occurs (it is a spatially restricted setting), and that multiply infected cells show a significantly higher rate of virus production than singly infected cells. We are in the process of examining this in more detail. We have now brought this example into the discussion.

“Page 11: Do we have any evidence that the initial growth phase is faster than exponential?”

**Author reply:** No, so far there is no evidence, but it also has not been looked for. We have now extensively discussed this. In the context of HIV, it is possible that the acceleration occurs relatively early and has been missed in data, or that other factors come into play that modify the dynamics, specifics of which are discussed now. We are also in the process of examining this in the context of the adenovirus system mentioned above. The point of the model was to make the opposite assumption compared to previous work that assumed no change in burst size in multiply infected cells and to examine how this affects the dynamics. This information can then be used to gain a better understanding of specific infections. In the case of HIV, the dependence of burst size on the number of viruses in the cell can be measured directly. If the burst size does go up, the dynamics explored here can be investigated specifically. If aspects such as growth acceleration cannot be observed in specifically designed experiments, this means that other factors are important that are not taken into account in standard virus dynamics models, and it can help us identify these factors.

*“Page 13: Discussing the effect of saturation, i.e., the value of ε, helps to gain better understanding of the results. Having 1000 target cells at the uninfected steady state and ε = 1, the (corrected?) saturation*$T/\left(\varepsilon +T\right)$*basically remains one for a wide range of target cell levels. This implies that initially there is hardly any target cell limitation, and that all virus particles can easily find uninfected target cells. Nevertheless this is the regime where you claim to have have the strongest impact of coinfection. Can this be intuitively explained?”*

**Author response:** We tried to do this. Essentially, the fraction of multiply infected cells is higher if ε is relatively small, so they contribute more strongly to the dynamics as the virus population grows. The fraction of multiply infected cells is very small if we have βTv (very larger ε in our model), and thus their contribution is less noticed and you only observe acceleration when addition of more viruses leads to a very large (unrealistic) increase in the viral replication rate (high g).

“Figures: what is the value of η ? Why not use parameter values that are somewhat realistic?”

**Author response:** The value of η has now been specified in all legends. Regarding parameter values, while we have discussed HIV a lot, this model is much more general and also is likely interesting with respect to other infections as well (perhaps more so), specifically the adenovirus example described above. HIV is discussed a lot because this is the system where multiple infection has been demonstrated experimentally in the most detailed way. However, it is also an infection that is characterized by many further complications that go beyond the basic virus dynamics model used here. We have added discussion to this effect. Using realistic parameter values will make most sense once a system is fully parameterized (and the model thus becomes truly predictive), as we are currently trying to do with the adenovirus system.

Reviewer 2 – Prof. Ruy M. Ribeiro

“This is a conceptual paper that proposes a simple model to raise interesting ideas about the effects of multiple infection of the same cell by HIV-1. The paper is well written and describes the research clearly. I have only a few comments.”

“In model 1 (and successive forms), it is not clear why the (1+epsilon) appears in the numerator. Or, saying it in another way, why was this form for the saturation of infection with T chosen? More usual forms would be epsilon/(T+epsilon) or even just 1/(epsilon+T), in which case than changing epsilon also implies changing beta. This probably does not make any difference for the analysis and results, but a short comment would be appreciated.”

**Author response:** We have added a short comment about this saturation term. Yes, a more usual form would be 1/(epsilon+T). The (1+epsilon) in the numerator was introduced for convenience such that increasing epsilon won’t significantly decrease the rate of infection.

“In the first line after model 2, you say that infected cells die with rate a_i I_i, but this is not reflected in the model nor is part of your analyses discussion. In fact, I think it would be good to say something more about the possibility of cells infected with more viruses, and thus producing more viruses, dying more rapidly.”

**Author response:** In this section, a_i I_i is a typo because we are discussing a model without increased viral replication in multiply infected cells. However, it should apply to the next section (Increased viral replication in multiply infected cells) and the reviewer correctly points out that this should be discussed. We have done this.

“In section 2.3(b), second paragraph, the authors discuss the initial growth rate of the virus and that under the current model it wouldn’t be a simple exponential. This is stressed again later in the Discussion. Something should be said that in general the exponential growth rate seems to decline at high viral loads, as the peak in primary infection is reached, and that there are not a lot of evidence to indicate that the exponential rate actually increases. Perhaps this should come together with the author’s argument that there not enough measurements in early infection. Another possibility is for the authors to give some more details regarding under what parameter regimens one could or could not see this increase in exponential growth rate.”

**Author response:** We agree. The acceleration should take place before growth slows down at the peak. However, there is so far no experimental documentation that the acceleration indeed takes place in HIV infection. We have now added more text to discuss this, to describe conditions that promote the occurrence of accelerated growth, and to describe possible experimental approaches to test the model.

“The last sentence before section 2.4 is not clear. Shouldn’t the infected cells accumulate in the I_n population? Why is it that in the figure all classes of infected cells go down with large beta?”

**Author response:** We do not plot I_n. In general, I_n is an artificial end of the multiple infection cascade that has to be assumed in an ODE framework like this. We have added a short explanation.

*“InEffect of target cell saturation, when discussing the role of epsilon, it would be good to see if for large values of epsilon, viral load can continue to increase for a very long time and the set-point viral load is much higher, perhaps unrealistically so. Also, in this page, weren’t the co-infection experiments in vitro? If so, it is not clear that they can be used to justify the point that the authors are making regarding low epsilon in vivo.”*

**Author response:** Yes, the experiments were in vitro, and we have now re-written it to reflect this and to be more cautious about what one can say about in vivo. Large values of epsilon do not lead to much higher virus loads in this model due to target cell limitation.

“In the figures for early infection, there doesn’t seem to be a well defined and pronounced peak; which is typically seen in the data. Is this because of the parameters chosen or the structure of the model? If the latter than this is an important issue.”

**Author response:** This is because of a combination of parameter choice and the lack of immune responses in the model. The model can show a more pronounced peak, but in the absence of immune responses, it can be difficult to account for a very pronounced decline in virus load following the peak, typically observed in in vivo data. We have added text to discuss this.

*“In* Figure 2*, where you show the “pro-virus” level, what does that correspond to in the model?”*

**Author response:** It corresponds to the number of viral genome copies across all cells. We have now defined it more clearly.

“The authors study the effect of treatment during primary infection, and show that its success depends, in this model, on the viral load at the start of treatment. Is this also true after the set-point viral load has been achieved – i.e., therapy at different levels of set-point viral load (presumably different individuals) could have implications for treatment outcome?”

**Author response:** Yes, it would apply to this as well. We have added a figure to demonstrate this.

Reviewer 3 – Prof. Marek Kimmel

“The paper identifies an interesting model of viral infection depending on the initial viral load and draws conclusions concerning multistability of infection under such assumptions. The model includes infection classes defined by the number of viral particles per cell.

I find the paper methodology insufficient in that it neither provides a direct comparison to data based on a particular viral infection, nor does it provide a mathematical analysis, which might characterize general types of behavior. Instead, the authors only list several types of infection where the model might apply and, on the other hand, compute some straightforward mathematical characteristics such as equilibrium solutions.

However, the stability of the equilibria does not seem to have been studied, not mentioning an analysis of basins of attraction. Also, it seems that the same qualitative behavior of the model might be obtained by replacing the specific formula listed on p. 8, with a more general monotonous relationship. I think the paper has some potential for being publishable, but definitely needs more work. In additon I think that the authors might decide if they wish to fit the data or pursue a qualitative analysis of solutions. Both aims are worthwhile, but maybe too much for a single paper.”

**Author response:** We agree that a detailed stability analysis would be desirable. However, this is very complicated. It is impossible to even write down the equilibria of the system where multiple infection leads to an increased burst size of infected cells. In a follow-up study, we are considering simplified models in order to study these phenomena on a more mathematical level, but this is rather complicated, will take some time, and is beyond the scope of this paper. For this paper, we tried to tackle this numerically, but even this turned out to be characterized by complications. We thus did not want to present it in the current paper but decided to wait until our ongoing work has yielded a clearer picture. The current paper presents several results that are interesting from a biological and virus dynamics point of view and we think that it is valuable to get this out. A detailed mathematical analysis, aimed at a different readership, will follow. We have added some discussion to the manuscript.

The same applies to the expression describing the rate of viral replication as a function of the number of resident viruses in cells. We tried an alternative expression, and results did not change on a qualitative level. However, this again needs to be analyzed more rigorously and generally, using axiomatic modeling approaches, and we are currently working on that. It is premature to present at this stage, and we aim to include this in the same study that examines these phenomena from a more mathematical point of view. Again, we have added some sentences to discuss this.

The most interesting biological system to apply this model to would be HIV infection, because multiple infection is well documented, and we have discussed this case quite extensively in the manuscript However, currently there are no data that show an accelerated virus growth during primary infection. There could be a number of reasons for this, and they are discussed in the revision. The aim of this paper was to show how the very basic virus dynamics can change if you assume that multiply infected cells have a higher bust size in the context of standard virus dynamics models. In particular, it is important to contrast this with a recent study that assumed multiply infected cells to have the same burst size as singly infected cells. Understanding the properties of models with different assumptions is an important contribution such that we can distinguish between hypotheses, or identify further mechanisms that need to be taken into account to explain and reconcile experimental data.

## Competing interests

The authors declare that they have no competing interest.

## Authors’ contributions

KWC analyzed the model. DNL and DW constructed the model and wrote the paper. All authors read and approved the final manuscript.

## Author information

KWC is a graduate student at University of California Irvine, studying mathematical models of virus infections. DNL is faculty at New York University and is an experimental HIV researcher. DW is faculty at University of California Irvine, and is a theoretical biologists studying the dynamics of viral infections.
